# High-efficacy targeting of colon-cancer liver metastasis with *Salmonella typhimurium* A1-R via intra-portal-vein injection in orthotopic nude-mouse models

**DOI:** 10.18632/oncotarget.12227

**Published:** 2016-09-23

**Authors:** Kei Kawaguchi, Takashi Murakami, Atsushi Suetsugu, Tasuku Kiyuna, Kentaro Igarashi, Yukihiko Hiroshima, Ming Zhao, Yong Zhang, Michael Bouvet, Bryan M. Clary, Michiaki Unno, Robert M. Hoffman

**Affiliations:** ^1^ AntiCancer, Inc., San Diego, California, USA; ^2^ Department of Surgery, University of California San Diego, San Diego, California, USA; ^3^ Department of Surgery, Graduate School of Medicine, Tohoku University, Sendai, Japan

**Keywords:** Salmonella typhimurium A1-R, tumor targeting, intra-portal vein injection, liver metastasis, colon cancer

## Abstract

Liver metastasis is the main cause of colon cancer-related death and is a recalcitrant disease. We report here the efficacy and safety of intra-portal-vein (iPV) targeting of *Salmonella typhimurium* A1-R on colon cancer liver metastasis in a nude-mouse orthotopic model. Nude mice with HT29 human colon cancer cells, expressing red fluorescent protein (RFP) (HT29-RFP), growing in the liver were administered *S. typhimurium* A1-R by either iPV (1×10^4^ colony forming units (CFU)/100 μl) or, for comparison, intra-venous injection (iv; 5×10^7^ CFU/100 μl). Similar amounts of bacteria were delivered to the liver with the two doses, indicating that iPV delivery is 5×10^3^ times more efficient than iv delivery. Treatment efficacy was evaluated by tumor fluorescent area (mm^2^) and total fluorescence intensity. Tumor fluorescent area and fluorescence intensity highly correlated (p<0.0001). iPV treatment was more effective compared to both untreated control and iv treatment (p<0.01 and p<0.05, respectively with iPV treatment with S. typhimurium arresting metastatic growth). There were no significant differences in body weight between all groups. The results of this study suggest that *S. typhimurium* A1-R administered iPV has potential for peri-operative adjuvant treatment of colon cancer liver metastasis.

## INTRODUCTION

Liver metastasis is the main cause of colon-cancer-related death [1]. In some cases, surgical resection of colon cancer liver metastasis is effective; however, the recurrence rate is extremely high [2]. The efficacy of adjuvant chemotherapy has been limited [3]. Therefore, development of effective treatment for colon cancer metastasis is needed.

The tumor-targeting *Salmonella typhimurium* A1-R (*S. typhimurium* A1-R), developed by our laboratory [4], is auxotrophic for Leu—Arg, which prevents it from mounting a continuous infection in normal tissues. *S. typhimurium* A1-R was effective against primary and metastatic tumors as monotherapy in nude mouse models of major cancers [5], including prostate [6, 7], breast [8–10], lung [11, 12], pancreatic [13–17], ovarian [18, 19] stomach [20], and cervical cancer [21], as well as sarcoma cell lines [22–25] and glioma [5, 26], all of which are highly aggressive tumor models. In addition, *S. typhimurium* A1-R was effective against patient-derived orthotopic models of pancreatic cancer [27, 28], sarcoma [25, 29–32] and melanoma [33].

In orthotopic mouse models, *S. typhimurium* A1-R, delivered iv, targeted liver metastases and significantly reduced their growth. The results of this previous study demonstrated the future clinical potential of *S. typhimurium* A1-R targeting of liver metastasis [34].

Regional chemotherapy of metastasis has resulted in higher levels of active 5-FU metabolites in the liver [35, 36]. Previous studies have demonstrated that 5-FU administered directly into the portal vein adjuvantly may decrease distant metastases [37, 38]. Chang, et al. [39] reported that peri-operative intraportal (iPV) chemotherapy combined with adjuvant chemotherapy was useful to prolong disease-free survival after primary tumor resection and decreased liver metastasis for stage II and III colon-cancer patients without liver metastasis.

Previously, we administered 5-FU, ip, 2 h before hepatic resection of the human colon tumors, with therapy continued postoperatively for 4 consecutive days. We termed this procedure neo-neoadjuvant chemotherapy. Neo-neoadjuvant therapy significantly prolonged animal survival compared with standard preoperative 5-FU neoadjuvant therapy, 5-FU post-operative adjuvant therapy, surgery alone, 5-FU without surgery, or the untreated control. When all animals with neoadjuvant 5-FU treatment had died, 70% of animals with neo-neoadjuvant treatment were still alive. Survival of mice treated with 5-FU without surgery, surgery alone, and adjuvant postoperative chemotherapy, was not significantly different from the untreated control group. Whereas 100% of animals in the control, 90% in the 5-FU alone, 70% in the surgery alone, 60% in the 5-FU adjuvant, and 40% in the neoadjuvant groups had metastases in the lymph nodes draining the liver, only 10% of animals in the neo-neoadjuvant group had metastases [40].

The present study evaluates the efficacy and safety of iPV of *S. typhimurium* A1-R on colon cancer liver metastasis in a nude-mouse orthotopic model.

## RESULTS AND DISCUSSION

### iPV injection is more effective for delivery of *S. typhimurium* A1-R to the liver then iv injection

**Figure 1:**
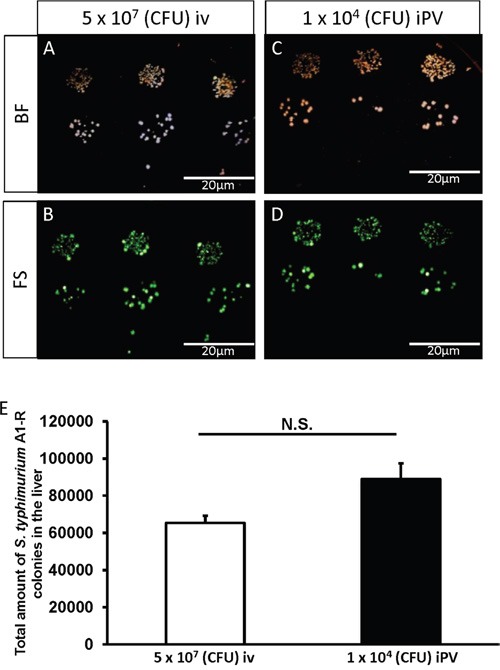
Culture of *S. typhimurium* A1-R from mouse liver **A-D**. Representative images of *S. typhimurium* A1-R colony formation: Bright field (BF) (A) and fluorescence (FS) (B) after intravenous (iv) injection of *S. typhimurium* A1-R (5 × 10^7^ CFU). BF (C) and FS (D) after intra-portal-vein (iPV) injection of *S. typhimurim* A1-R (1 × 10^4^ CFU). The liver was minced and mixed with PBS and was seeded on LB-Agar with serial dilution in triplicate. Fluorescent *S. typhimurium* A1-R colonies were observed with the OV100 Small Animal Imaging System (Olympus Corp, Tokyo, Japan). **E**. *S. typhimurium* A1-R colony number in the liver after iv and iPV injection.

Two days after injection of *S. typhimurium* A1-R (iv: 5×10^7^ CFU/100 μl; iPV: 1×10^4^ CFU/100 μl) to mice without liver metastasis, the liver was removed and cultured on Luria-Bertani (LB) agar. The presence of *S. typhimurium* A1-R was confirmed by bright-field and GFP-expressing colony formation 24 hours after culture (Figure 1A-1D). There was no significant difference in colony formation between iv injection of 5×10^7^ CFU/100 μl and iPV injection of 1×10^4^ CFU/100 μl *S. typhiurium* A1-R. These results showed that iPV injection was 5×10^3^ times more effective for delivery of *S. typhiurium* A1-R to the liver than iv injection (Figure 1E).

### Efficacy of *S. typhimurium* A1-R iPV injection on liver metastasis

**Figure 2:**
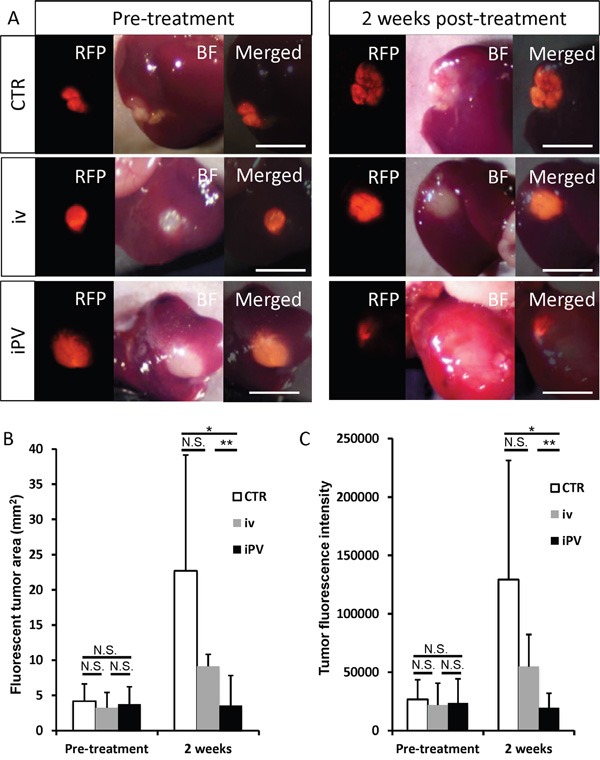
Efficacy of *S. typhimurium* A1-R on HT29-RFP liver metastasis **A**. Pre-treatment and 14 days post-treatment of *S. typhimurium* A1-R: iv, 5 × 10^7^ CFU/100μ; iPV, 1 × 10^4^ CFU/100 μl. No treatment control (CTR). **B-C**. Bar graphs show the tumor fluorescent area (mm^2^) (B) and fluorescence intensity (C) at day 14. Scale bars: 5 mm.

**Figure 3:**
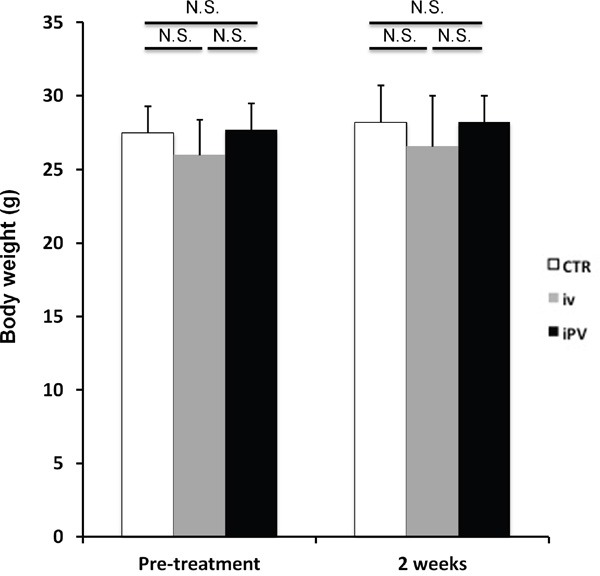
Safety evaluation of *S. typhimurium* A1-R therapy Bar graph shows body weight in each group at pre-treatment and on day 14. There were no significant differences between the treated groups and control.

The fluorescent area and total fluorescence intensity of HT29 growing in the liver were measured on day 14 (Figure 2A) after iPV or iv injection of *S. typhimurium* A1-R. iPV delivery of *S. typhiurium* A1-R significantly suppressed growth of HT29 in the liver compared to both the untreated-control group and iv treatment group (p<0.01, p<0.05, respectively) (Figure 2B, 2C), with IVP formatting arresting metastatic growth. There was no significant body-weight difference between the groups (Figure 3).

This is the first study to administer *S. typhimurium* A1-R via the iPV route to the liver directly. Most importantly, the treatment efficacy of iPV injection was significantly better than iv injection on HT29 cells growing in the liver.

Intra-operative therapy could be useful to decrease the risk of recurrence after cancer resection and could increase the surgical cure rate for colon-cancer liver metastasis patients. It has been reported that peri-operative intra-portal chemotherapy combined with adjuvant chemotherapy prolonged disease-free survival after primary tumor resection and decreased liver metastasis for stage II and III colon cancer patients, without liver metastasis [39].

These results are consistent with our previous studies which showed peri-operative “neo-neoadjuvant chemotherapy” was effective against colon-cancer liver metastasis in orthotopic nude mouse models [40].

**Figure 4:**
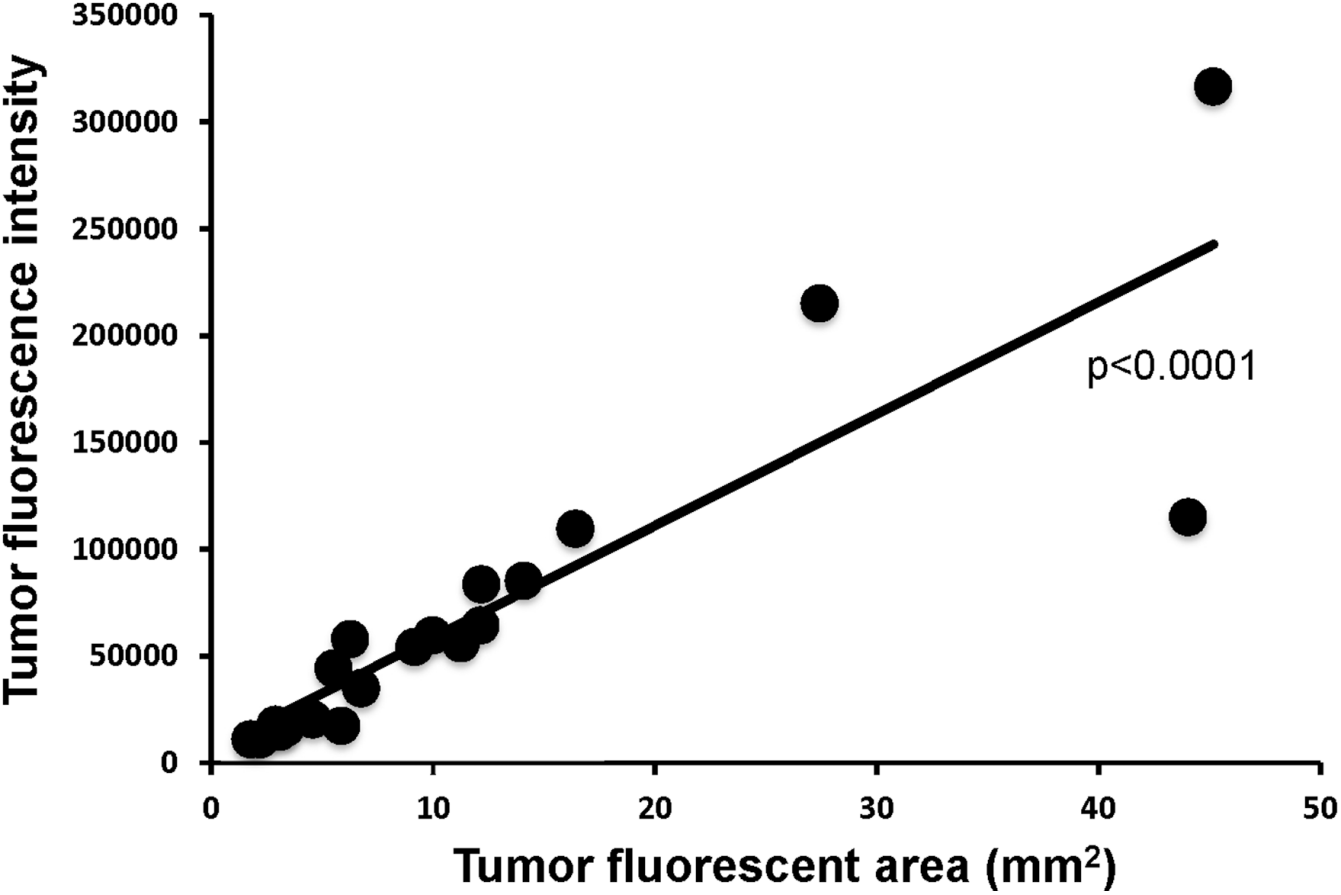
Correlation of tumor fluorescent area in tumor fluorescence intensity Tumor area significantly correlated with fluorescence intensity (p<0.0001).

In the present study, we demonstrated a strong correlation between tumor fluorescence area and fluorescence intensity (p<0.0001) (Figure 4). Fluorescence intensity of tumors could be a readily-measured simple parameter for determination of efficacy.

### Intravital color-coded imaging of bacterial targeting of liver metastasis

**Figure 5:**
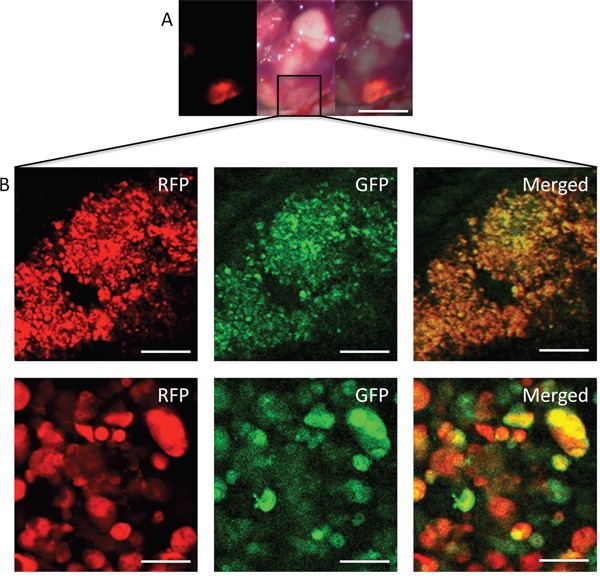
Intravital imaging of *S. typhimurium* A1-R-GFP targeting HT29-RFP liver metastasis via iPV injection *S. typhimuriumu* A1-R-GFP was visualized targeting the HT29-RFP liver metastases at day 14 after iPV injection. **A**. Liver metastases were visualized with the OV100 by fluorescence (left); brightfield (center) and merge (right). **B**. Confocal imaging with the FV1000 demonstrated *S. typhimuriumu* A1-R-GFP targeting the HT29-RFP liver metastasis at the cellular level. Scale bars: (A) 5 mm; (B) Upper panels: 50 μm; Lower panels: 12.5 μm.

Intravital color-coded imaging visualized *S. typhimurium* GFP targeting the HT29-RFP liver metastasis cells 14 days after iPV injection. The GFP-expressing bacteria can be seen growing in the HT29-RFP cells (Figure 5).

The present study develops a new concept of iPV delivery of tumor-targeting *S. typhimurium* A1-R to treat colon-cancer liver metastasis, the results of which are very promising. Bacterial therapy of cancer, which was first line for sarcoma and other cancers in the early part of the last century [5], is now having an exciting resurgence, including clinical trials [41].

Previously developed concepts and strategies of highly selective tumor targeting can take advantage of bacterial targeting of tumors [42–46].

## MATERIALS AND METHODS

### Cell line

HT29 human colon cancer cells expressing RFP [34, 47–49] were maintained in RPMI 1640 medium (Invitrogen, Carlsbad, CA, USA) supplemented with heat-inactivated 10% fetal bovine serum (FBS) (Gemini Biologic Products, Calabasas, CA), 2 mM glutamine, 100 units/ml penicillin, 100 μg/ml streptomycin and 0.25 μg/ml amphotericin B (Life Technologies, Inc., Grand Island, NY). The cells were incubated at 37°C in 5% CO_2_.

### Mice

Athymic *nu/nu* nude mice (AntiCancer Inc., San Diego, CA), 4–6 weeks old, were used in this study. All mouse surgical procedures and imaging were performed with the animals anesthetized by subcutaneous injection of a ketamine mixture (0.02 ml solution of 20 mg/kg ketamine, 15.2 mg/kg xylazine, and 0.48 mg/kg acepromazine maleate). All animal studies were conducted in accordance with the principles and procedures outlined in the National Institutes of Health Guide for the Care and Use of Animals under Assurance Number A3873-1.

### Establishment of liver metastases

HT29-RFP cells were harvested by trypsinization and washed twice with serum-free medium. HT29-RFP cells (5×10^5^ in 50 μl serum-free medium with 50% Matrigel) were injected into the superior and inferior pole of the spleen in nude mice. Three weeks after injection, experimental liver metastases were established.

### Surgical orthotopic implantation of liver metastasis

Experimental liver metastases, as described above, were resected and cut into blocks (8 mm^3^). A single tumor block was orthotopically implanted into the left lobe of the liver in nude mice. Four weeks later, orthotopic liver metastasis were observed at the implanted site by RFP expression.

### Preparation of *S. typhimurium* A1-R

GFP-expressing *S. typhimurium* A1-R bacteria (AntiCancer Inc.,) were grown overnight on LB medium (Fisher Sci., Hanover Park, IL, USA) and then diluted 1:10 in LB medium. Bacteria were harvested at late-log phase, washed with PBS, and then diluted in PBS [6–8].

### iPV injection

The anesthetized mice were placed in a supine position. After disinfecting the skin in the area of surgery, a median abdominal incision was performed followed by mobilization of the duodenum to identify the portal vein. *S. typhimurium* A1-R (100 μl) was injected into the portal vein using a 31 G needle. After removal of the needle, bleeding was stopped by gently pressing the puncture site with a cotton swab. After injection, the intestine was repositioned and the abdominal wall was closed with non-absorbable sutures.

### Targeting of the liver with *S. typhimurium* A1-R by iPV or iv routes

Nude mice without tumor were injected with S. typhimurium A1-R (iPV: 1×10^4^ CFU; iv: 5×10^7^ CFU). On day 2 after injection, the liver was resected, minced, and mixed with PBS. The minced liver was placed on LB agar to identify *S. typhimurium* A1-R. Fluorescent *S. typhimurium* A1-R bacteria were observed with the OV100 Small Animal Imaging System (Olympus Corp., Tokyo, Japan).

### Imaging of tumor-targeted bacteria

The OV100 (Olympus) variable-magnification fluorescence imager [50] was used to image colonies of *S. typhimurium* A1-R from resected tumors. The FV1000 confocal microscope (Olympus) [51] was used to image resected tumors for the presence of *S. typhimurium* A1-R-GFP.

### Efficacy of *S. typhimurium* A1-R on liver metastasis

Four weeks after orthotopic implantation of HT- 29-RFP to the liver, 21 mice were randomized into 3 groups: untreated control group (*n* = 7); *S. typhimurium* A1-R iv treatment group (*S. typhimurium* A1-R, 5 × 10^7^ CFU/body, iv, *n* = 7); and *S. typhimurium* A1-R iPV treatment group (*S. typhimurium* A1-R, 1 × 10^4^ CFU/body, iPV, *n* = 7). The left lobe of the liver with metastasis was exposed before (at day 1) and after treatment (at day 15) for imaging with the OV100. The tumor fluorescent area and total fluorescence intensity were analyzed with UVP software (UVP, Upland, CA). Treatment efficacy in each mouse was evaluated by fluorescent area and total fluorescence intensity at day 15 compared to at the beginning of treatment. Liver metastasis in the *S. typhimurium* A1-R iPV treatment group was also imaged with the FV1000 confocal microscope at day 15 to observe *S. typhimurium* A1-R-GFP targeting the RFP-expressing HT29 liver metastasis at the cellular level.

### Statistical analysis

JMP version 11.0 was used for all statistical analyses. Significant differences for continuous variables were determined using the Mann-Whitney *U* test. Bar graphs expressed average values, and error bar showed SE. A probability value of *P* ≤ 0.05 was considered statistically significant.
